# Guaranteed resonance enclosures and exclosures for atoms and molecules

**DOI:** 10.1098/rspa.2014.0488

**Published:** 2014-11-08

**Authors:** Sabine Bögli, B. Malcolm Brown, Marco Marletta, Christiane Tretter, Markus Wagenhofer

**Affiliations:** 1 Mathematisches Institut, Universität Bern, Alpeneggstrasse 22, 3012 Bern, Switzerland; 2 School of Computer Science, Cardiff University, 5 The Parade, Cardiff CF24 3AA, UK; 3 School of Mathematics, Cardiff University, 21–23 Senghennydd Road, Cardiff CF24 4AG, UK; 4 Mathematisches Institut, Universität Bern, Sidlerstrasse 5, 3012 Bern, Switzerland; 5 Matematiska institutionen, Stockholms universitet, 10691 Stockholm, Sweden; 6 Im Gallmoos 11, 93128 Regenstauf, Germany

**Keywords:** resonance, eigenvalue, complex potential, interval arithmetic

## Abstract

In this paper, we confirm, with absolute certainty, a conjecture on a certain oscillatory behaviour of higher auto-ionizing resonances of atoms and molecules beyond a threshold. These results not only definitely settle a more than 30 year old controversy in Rittby *et al.* (1981 *Phys. Rev. A* **24**, 1636–1639 (doi:10.1103/PhysRevA.24.1636)) and Korsch *et al.* (1982 *Phys. Rev. A* **26**, 1802–1803 (doi:10.1103/PhysRevA.26.1802)), but also provide new and reliable information on the threshold. Our interval-arithmetic-based method allows one, for the first time, to *en*close and to *ex*clude resonances with guaranteed certainty. The efficiency of our approach is demonstrated by the fact that we are able to show that the approximations in Rittby *et al.* (1981 *Phys. Rev. A* **24**, 1636–1639 (doi:10.1103/PhysRevA.24.1636)) *do* lie near true resonances, whereas the approximations of higher resonances in Korsch *et al.* (1982 *Phys. Rev. A* **26**, 1802–1803 (doi:10.1103/PhysRevA.26.1802)) do *not*, and further that there exist two new pairs of resonances as suggested in Abramov *et al.* (2001 *J. Phys. A* **34**, 57–72 (doi:10.1088/0305-4470/34/1/304)).

## 1. Introduction

Reliable and precise information on the location of resonances is very hard to obtain. While numerical approximations are widely used in physics, so far there has been no way to show that they produce results *near*, or *not near*, true resonances. The reason is that computations of complex eigenvalues in the presence of continuous spectrum are not backed up by any convergence results. This paper presents a new method that, for the first time, permits one to locate resonances with absolute certainty and high accuracy and, at the same time, to show that numerical approximations *fail* to lie near true resonances. We provide new and reliable information on the oscillatory behaviour of the real parts of certain resonance strings and on the threshold beyond which it occurs.

The key ingredient in our method is interval arithmetic. It allows us to carry out every computational step with absolute accuracy by operating on intervals rather than on numbers. Remarkably, this theoretical idea has had convincing impact in different practical physical applications recently: to control the stability of difficult nonlinear systems in robotics to navigate a sailboat autonomously over a distance of 100 km (see [1]); to perform rigorous global optimization of impulsive planet-to-planet transfer (see [2]) or to rigorously govern the long-term stability in particle accelerators (see [3]).

In this paper, we demonstrate the efficacy of interval approaches for the computation of resonance enclosures and exclosures with absolute certainty. The power of our method is substantiated by the fact that it can be applied to definitely settle a more than 30 year old controversy in [4,5] which could not be resolved by any other method before.

In connection with auto-ionizing resonances of atoms and molecules lying above a ionization threshold, Moiseyev *et al.* [6] studied resonances of the Sturm–Liouville problem

1.1 $$-y^{\prime \prime }(x)+\frac{2\mu }{\hbar^{2}}((0.5x^{2}-0.8)\;e^{-x^{2}/10}+0.8-\epsilon )y(x)=0,\;x\in R.$$

A first resonance was suggested to lie near 2.124−0.0185*i* (with $\mu /\hbar^{2}$ set to 1); moreover, one bound state was proposed to lie near 0.5. The resonance was found by complex scaling of the self-adjoint Hamiltonian and approximation using a variational principle with 10 real Gauss-type basis functions for the scaled Hamiltonian. Because the latter is no longer self-adjoint, the authors pointed out that further exploration is needed to obtain information on the true position of a nearby resonance.

In the two subsequent papers [4] and the more detailed version [7], Rittby *et al.* combined complex scaling with some Weyl-type analysis and numerical integration methods to compute 44 resonance approximations, including approximations for the first resonance and bound state suggested in [6]; the second resonance therein was further studied by Engdahl & Brändas in [8] by computing lower bounds for norms of Riesz projections. The main conclusion of [4,7] is that there exists a complex threshold *ϵ*_thresh_ with Re(*ϵ*_thresh_)>0, Im(*ϵ*_thresh_)<0 such that all resonance approximations of (1.1) satisfy Re(*ϵ*)≤Re(*ϵ*_thresh_)∼4.68 and beyond this threshold, i.e. for Im(*ϵ*)<Im(*ϵ*_thresh_)<0, their real parts exhibit a certain oscillatory behaviour.

Shortly after the publication of [4] and the submission of [7], Korsch *et al.* announced in [5], comment that they had computed a different set of resonance approximations beyond the threshold which did not exhibit any oscillatory behaviour, whereas their earlier computations of lower resonances in [9] had not shown such a disagreement. They used a complex-rotated Milne method and they believed to have backed up their computations by some WKB approximations. Korsch *et al.* concluded that the results of Rittby *et al.* for higher resonances were incorrect; they conjectured this might be due to numerical instabilities or to the too limited range 0<*θ*<*π*/4 of angles in the complex scaling method in [4,7].

In an immediate reply (see [10], reply to comment), Rittby *et al.* [5] defended their results and attributed the discrepancies of the results to wrongly chosen outgoing boundary conditions. They argued that the asymptotic solutions of the complex Riccati equation associated with (1.1) undergo a dramatic change when *θ* passes the critical value *θ*_crit_=*π*/4 of the potential in (1.1) and hence the rotation angle *θ*=50° used by Korsch *et al.* was too large. Because of this and the stability of the computations in [4,7] against variations of the rotation angle *θ*, Rittby *et al.* [4,7] believed to have found approximations to true resonances. About 10 years later, Andersson corroborated the arguments and conclusions of Rittby *et al.* by a careful multiple-transition point WKB analysis and explained the failure of the complex-rotated Milne method of Korsch *et al.* by semi-classical theory in [11].

Almost 20 years after the 1982 dispute, the resonance problem (1.1) was studied as an example in two papers in the mathematical literature. In [12], for more general classes of exponentially decaying potentials, Brown *et al.* developed a resonance-finding procedure for resonances close to points of spectral concentration on the real axis. This method relies on analytic continuation of the Weyl–Titchmarsh function rather than on complex scaling and was first used by Hehenberger *et al.* [13] in numerical computations for the Stark effect. As an example, Brown *et al.* computed approximations to the first three resonances of (1.1) which were very close to the ones found in [7]; note that $\mu /\hbar^{2}=1$ in [7] and that the potential *q* and spectral parameter λ in [12] are related to the potential *V* and spectral parameter *ϵ* in (1.1) by

$$q(x)=(x^{2}-1.6)\;e^{-x^{2}/10}=\frac{\hbar^{2}}{\mu }V(x)-1.6,\;\lambda =\frac{2\mu }{\hbar^{2}}(\epsilon -0.8).$$

Not long after, Abramov *et al.* [14] proved some global analytical bounds for resonances for various classes of potentials. They combined complex scaling with operator theoretic techniques such as numerical ranges and Birman–Schwinger type arguments. Moreover, for the particular case of (1.1), they also performed numerical computations. The analytical results in [14] supported the conjecture of Rittby *et al.* that a wrong asymptotic boundary condition was used by Korsch *et al.* [5]. The numerical results of [14] reproduced the resonances found in [4,7] and they suggested three pairs of additional resonances. Each pair consists of an even and an odd resonance so close to each other that they could not be computed accurately. These new resonance pairs may be related to the oscillatory behaviour of the real parts; because two of these pairs satisfy −9.57∼Im(*ϵ*_thresh_)<Im(*ϵ*)<0.

As it was rightly put in [14], none of the above methods for finding resonances can be used *to locate them accurately, but there is clear evidence that they exist*. Moreover, none of these methods allows for a proof that a numerically computed candidate for a resonance is *not* near any true resonance.

The method presented here permits us to settle both questions definitely and adds new information on the threshold beyond which oscillatory behaviour of the real parts of resonances occurs. We prove that the 44 numerical approximations of resonances from [4,7] do lie near true resonances and that the numerical approximations labelled 16–28 in [5] do *not* lie near true resonances. Moreover, we prove that two of the additional pairs of resonances conjectured in [14] do exist. Our provably correct computations are based on a combination of two key tools, the argument principle on the analytic side and interval arithmetic on the computational side.

Briefly, our approach is as follows. By means of complex scaling *x*→e^i*θ*^*x* with *θ*∈[0,*π*/4), the resonances *ϵ* of (1.1) are given in terms of the eigenvalues *z*=*e*^2i*θ*^(2*ϵ*−1.6) of a Sturm–Liouville problem on R with complex potential. These eigenvalues can be characterized as the zeros of an analytic function Δ. Hence, their number in a rectangle $R_{0}$ can be counted by means of the argument principle. On the other hand, we can compute the contour integral in the argument principle in interval arithmetic, using a code based on the software library VNODE developed by Nedialkov *et al*. [15]. Roughly speaking, this means that all computations, from adding numbers up to integration, amount to working with two-sided estimates; e.g. the sum of two real numbers *a*∈[*a*_1_,*a*_2_] and *b*∈[*b*_1_,*b*_2_] is the interval [*a*_1_+*b*_1_,*a*_2_+*b*_2_] which is guaranteed to contain *a*+*b* (see [16], §2 for a more detailed description). If we obtain that

1.2 $$\frac{1}{2\pi i}\int_{R_{0}}\frac{\Delta^{\prime }(z)}{\Delta (z)}\;dz\in [c_{1},c_{2}]\;\text{and}\;[c_{1},c_{2}]\cap N_{0}=\{n_{0}\},$$

then there are precisely *n*_0_ eigenvalues of the complex-scaled Hamiltonian in the rectangle $R_{0}$ and hence precisely *n*_0_ resonances in the rotated rectangle $e^{-2i\theta }R_{0}$.

Our method is the first, in both physical and mathematical literature, that accomplishes the following three different tasks:

1. Enclose resonances with prescribed accuracy, by choosing the size of the rectangle accordingly small and achieving *n*_0_=1.
2. Exclude resonances in certain rectangles by achieving *n*_0_=0.
3. Check if the number of resonances in a rectangle of arbitrary size computed with non-reliable methods is correct by checking if it coincides with *n*_0_.

## 2. Complex scaling and lack of analytic information

There are various mathematical definitions of resonances and different methods to study them; for details, we refer to the comprehensive review articles by Simon [17], Siedentop [18] and Harrell [19]. Here, we use the method of complex scaling where resonances are characterized as eigenvalues of certain non-self-adjoint Schrödinger operators.

As an example, we consider the spectral problem (1.1), with $\mu /\hbar^{2}=1$ for the sake of simplicity. If we set λ:=2*ϵ*−1.6, it is easy to see that (1.1) is equivalent to the spectral problem

2.1 $$-y^{\prime \prime }(x)+(x^{2}-1.6)\;e^{-x^{2}/10}y(x)-\lambda y(x)=0,\;x\in R,$$

for the linear operator *L* in the Hilbert space $L_{2}(R)$ given by

$$\begin{array}{rl} D(L) & :=W_{2}^{2}(R):=\{y\in L_{2}(R):y^{\prime },y^{\prime \prime }\in L_{2}(R)\},\\ \left(Ly)(x\right) & :=-y^{\prime \prime }(x)+(x^{2}-1.6)\;e^{-x^{2}/10}y(x); \end{array}$$

note that $W_{2}^{2}(R)$ is the closure of $C_{0}^{\infty }(R)$ with respect to the norm of $W_{2}^{2}(R)$ given by $∥y{∥}_{2,2}:={\left(∥y{∥}_{2}^{2}+∥y^{\prime }{∥}_{2}^{2}+∥y^{\prime \prime }{∥}_{2}^{2}\right)}^{1/2},$ where *y*′, *y*′′ denote the weak derivatives and ∥⋅∥_2_ denotes the norm of $L_{2}(R)$ ([20], ch. V).

According to the method of complex scaling ([21,22], [23], §5 and also [24]), for every *θ*∈[0,*π*/4), the spectral problem (2.1) is equivalent to the spectral problem for the operator *H*_*θ*_ in $L_{2}(R)$ given by $D(H_{\theta })=W_{2}^{2}(R)$ and

2.2 $$(H_{\theta }y)(x):=-y^{\prime \prime }(x)+q_{\theta }(x)y(x)=zy(x),\;x\in R,\;z:=e^{2i\theta }\lambda ,$$

with complex-valued potential

2.3 $$q_{\theta }(x):=e^{2i\theta }(e^{2i\theta }x^{2}-1.6)e^{-e^{2i\theta }x^{2}/10},\;x\in R.$$

Hence, *z* is an eigenvalue of (2.2) if and only if λ=*e*^−2i*θ*^*z* is a resonance of (2.1) or, equivalently, if *ϵ*=(λ+1.6)/2=(*e*^−2i*θ*^*z*+1.6)/2 is a resonance of (1.1).

Because *q*_*θ*_ is even, the spectral problem (2.2) for the operator *H*_*θ*_ is equivalent to the two spectral problems

2.4 $$-y^{\prime \prime }(x)+q_{\theta }(x)y(x)=zy(x),\;x\in [0,\infty ),\;y(0)=0$$

and

2.5 $$-y^{\prime \prime }(x)+q_{\theta }(x)y(x)=zy(x),\;x\in [0,\infty ),\;y^{\prime }(0)=0,$$

for the operators $H_{\theta }^{D}$ and $H_{\theta }^{N}$ induced by the differential expression *τ*_*θ*_*y*:=−*y*′′+*q*_*θ*_*y* in $L_{2}([0,\infty ))$ with Dirichlet and with Neumann boundary condition, respectively. This was proved in [12], §5 using the Weyl–Titchmarsh function. For eigenvalues, this follows from the following elementary argument.

If $z_{0}\in C$ is an eigenvalue of (2.2) with eigenfunction $y_{0}\in D(H_{\theta })\subset L_{2}(R)$, then, by (2.3), the function ${\tilde{y}}_{0}$ given by ${\tilde{y}}_{0}(x):=y_{0}(-x)$ is an eigenfunction as well. Because $y_{0}(0)={\tilde{y}}_{0}(0)$, the functions *y*_0_, ${\tilde{y}}_{0}$ must be linearly dependent. The particular form of ${\tilde{y}}_{0}$ implies that ${\tilde{y}}_{0}=\gamma y_{0}$ with *γ*=±1. Because $y_{0}\in W_{2}^{2}(R)\subset C^{1}(R)$, the continuity of *y*_0_ and *y*_0_′ in 0 yields that *y*_0_′(0)=0 if *γ*=1 and *y*_0_(0)=0 if *γ*=−1. Hence, $y_{0}{|}_{\left[0,\infty \right)}$ is either an eigenfunction of (2.4) or of (2.5). Vice versa, if $z_{0}\in C$ is an eigenvalue of (2.4) with eigenfunction $y_{0}\in D(H_{\theta }^{D})\subset L_{2}([0,\infty ))$, we obtain an eigenfunction $y_{0}\in D(H_{\theta })\subset L_{2}(R)$ of (2.2) at λ_0_ by setting *y*_0_(*x*):=−*y*_0_(−*x*), x∈(−∞,0); if $z_{0}\in C$ is an eigenvalue of (2.5), we set *y*_0_(*x*):=*y*_0_(−*x*), x∈(−∞,0).

Because the potential *q*_*θ*_ is complex-valued and hence all the above operators *H*_*θ*_ along with $H_{\theta }^{D}$, $H_{\theta }^{N}$ are no longer self-adjoint, numerical approximations of eigenvalues—and hence of resonances—are prone to be unstable. Examples for such instabilities may be found in [23] for resonances, but they occur already for eigenvalues of matrices (see e.g. [16] for the famous Godunov matrix).

Analytic bounds for resonances are commonly based on numerical range estimates for each complex-scaled problem (2.2) with *θ*∈[0,*π*/4) (comp. [14]). For the set of resonances of (1.1), we obtain the following result.

**Theorem 2.1**

*The resonances of (*1.1*) in the sector* −π/2<arg⁡ϵ≤0 *are contained in the closed convex set
*

C:=⋂θ∈[0,π/4){ϵ∈C:Re(ϵ)sin⁡(2θ)+Im(ϵ)cos⁡(2θ)≤0.5a+(θ)+0.8sin⁡(2θ)}

*where* a+(θ):=supx∈[0,∞)Im(qθ(x)) *for θ∈[0,π/4) with
*

Im(qθ(x))=e−cos⁡(2θ)x2/10(x2sin(4θ−sin⁡(2θ)x210)−1.6sin(2θ−sin⁡(2θ)x210)).

**Proof.**

The set C is closed and convex being the intersection of closed half-planes. Because *a*_+_(*θ*)≥0 and hence $a_{+}(\theta )/(2\sin(2\theta ))+0.8\geq 0.8$, it follows that C contains all ϵ∈C with 0<Re(*ϵ*)≤0.8 and Im(*ϵ*)≤0.

Thus, it is sufficient to show that every resonance $\epsilon_{0}\in C$ with Re(*ϵ*_0_)>0.8, Im(*ϵ*_0_)≤0 belongs to C. For every *θ*∈[0,*π*/4], the point λ_0_:=2*ϵ*_0_−1.6 lies in the sector −π/2<arg⁡λ≤0 and is an eigenvalue of the operator ${\tilde{H}}_{\theta }:=e^{-2i\theta }H_{\theta }$ given by

$$D({\tilde{H}}_{\theta })=W_{2}^{2}(R),\;{\tilde{H}}_{\theta }y=e^{-2i\theta }(-y^{\prime \prime }+q_{\theta }y).$$

Because the numerical range of a linear operator contains all eigenvalues, we obtain

$$\lambda_{0}\in W({\tilde{H}}_{\theta }):=\{({\tilde{H}}_{\theta }y,y):y\in D({\tilde{H}}_{\theta }),∥y∥=1\},\;\theta \in \left[0,\frac{\pi }{4}\right).$$

If we note that $q_{\theta }(R)=q_{\theta }([0,\infty ))$ and, in addition to *a*_+_(*θ*), we define

a−(θ):=infx∈[0,∞) Im(qθ(x)),b−(θ):=infx∈[0,∞) Re(qθ(x)),

then it is easy to see that, for *θ*∈[0,*π*/4),

W(H~θ)⊂e−2iθ{z∈C:a−(θ)≤Im(z)≤a+(θ),b−(θ)≤Re(z)}.

In particular, every resonance λ_0_ of *L* with −π/2<arg⁡λ≤0 satisfies

λ0∈⋂θ∈[0,π/4){λ∈C:Re(λ)sin⁡(2θ)+Im(λ)cos⁡(2θ)≤a+(θ)}.

 ▪

**Figure 1.**
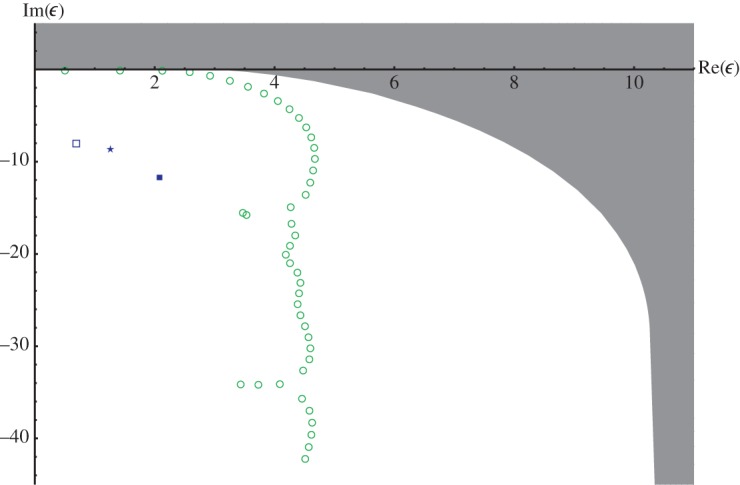
Resonance-free region in theorem 2.1 (grey-shaded), approximate resonance strings of Rittby *et al.* (circles) and Abramov *et al.* (squares and star). (Online version in colour.)

Figure 1 shows that the only available analytic information is much too coarse to judge the validity or non-validity of resonance approximations. Therefore, it is necessary to employ a method yielding both guaranteed and much more accurate enclosures and exclosures for eigenvalues of non-self-adjoint eigenvalue problems.

## 3. Eigenvalue enclosures for complex-valued potentials

The algorithm we use to establish guaranteed eigenvalue enclosures was developed and described in detail in [25,16]. Briefly, it consists of the following two steps. For the sake of simplicity, we consider the Dirichlet problem (2.4); the approach to the Neumann problem (2.5) is completely analogous.

*Step* *A*. *Solving a truncated problem with guaranteed error bounds*. In order to truncate problem (2.4), we restrict the potential *q*_*θ*_ to an interval [0,*X*] and set it equal to 0 on (X,∞). The unique (up to scalar multiplication) solution of −*y*′′=*zy* in $L_{2}([X,\infty ))$ is $\exp(-\sqrt{-z}x)$ for Re−z>0. Hence, the problem on [0,*X*], we have to solve is

3.1 $$\begin{array}{rl} & -y^{\prime \prime }(x)+q_{\theta }(x)y(x)=zy(x),\;x\in [0,X]\\ \text{and}\; & y(0)=0,\;y^{\prime }(X)=-\sqrt{-z}y(X). \end{array}\}$$

The eigenvalues of this regular boundary value problem can be characterized as the zeros of an analytic function Δ and may thus be counted and found by means of the argument principle.

The algorithms for the calculation of the analytic function Δ and for the contour integral over a chosen starting box $R_{0}\subset C$ are performed in interval arithmetic, i.e. with guaranteed error bounds. Having achieved (1.2), we obtain a box that contains a certain number *n*_0_ of eigenvalues of the truncated problem (3.1). Repeating this procedure by suitably subdividing the box $R_{0}$, we may finally arrive at a box $R_{Z}$ of desired precision *ε*_*Z*_ that contains exactly one eigenvalue *z*_*trunc*_.

*Step* *B*. *Use Levinson asymptotics to enclose the eigenvalues of problem* (2.4). If *y*_2_(⋅,*z*) is the unique (suitably normalized) solution of the differential equation in (2.4) belonging to $L_{2}([0,\infty ))$ for z∈C∖[0,∞), then *z*_true_ is an eigenvalue of (2.4) if and only if *y*_2_(0,*z*_true_)=0. Levinson's theorem (see e.g. [25], theorem 3.3) shows that

3.2 $$y_{2}(x,z)=\exp(-\sqrt{-z}x)(1+\eta (x)),\;|\eta (x)|\leq \frac{\alpha_{X,\theta }}{1-\alpha_{X,\theta }},\;\alpha_{X,\theta }:=\int_{X}^{\infty }|q_{\theta }(x)|\;dx,$$

for all *X*≥0 such that *α*_*X*,*θ*_<1. Hence, if [E]⊂R is an interval with

$$\left[1-\frac{\alpha_{X,\theta }}{1-\alpha_{X,\theta }},1+\frac{\alpha_{X,\theta }}{1-\alpha_{X,\theta }}\right]\subset [E],$$

and [*y*_2_(⋅,*z*)] is an interval-valued solution of the truncated problem on [0,*X*] satisfying the interval-valued initial conditions

$$y(X,z)\in [E]\exp(-\sqrt{-z}X),\;y^{\prime }(X,z)\in -[E]\sqrt{-z}\exp(-\sqrt{-z}X),$$

then *y*_2_(0,*z*)∈[*y*_2_(0,*z*)]. By means of the interval arithmetic argument principle already used in step A, we now obtain enclosures for the zeros of *y*_2_(0,*z*), and hence for the eigenvalues *z*_true_ of (2.4) of desired precision.

For the above-described method, several parameters have to be provided; in particular, the length *X* of the truncated interval has to be determined such that *α*_*X*,*θ*_<1. To this end, we note that

$$|q_{\theta }(x)|=|e^{2i\theta }x^{2}-1.6|\;e^{-\cos(2\theta )x^{2}/10}\leq x^{2}e^{-\cos(2\theta )x^{2}/10}\;\text{if }\cos(2\theta )\geq \frac{0.8}{x^{2}}$$

and that [26, 7.1.13]

$$\int_{x_{0}}^{\infty }e^{-x^{2}}\;dx\leq e^{-x_{0}^{2}}\frac{1}{x_{0}+\sqrt{x_{0}^{2}+4/\pi }},\;x_{0}\geq 0.$$

Integrating by parts and substituting $t=\sqrt{a}x$, we obtain, for *a*≥0,

$$\begin{array}{rl} \int_{X}^{\infty }x^{2}\;e^{-ax^{2}}\;dx & =\frac{1}{a}X\;e^{-aX^{2}}+\frac{1}{a\sqrt{a}}\int_{\sqrt{a}X}^{\infty }e^{-t^{2}}\;dt\\ & \leq \frac{1}{a}\;e^{-aX^{2}}\left(X+\frac{1}{aX+\sqrt{a^{2}X^{2}+4a/\pi }}\right)\\ & \leq \frac{1}{a}\;e^{-aX^{2}}\left(X+\frac{1}{2aX}\right). \end{array}$$

Hence, for all X∈(0,∞), *θ*∈[0,*π*/4) with $\cos(2\theta )\geq 0.8/x^{2}$, we can estimate

3.3 $$\alpha_{X,\theta }\leq \int_{X}^{\infty }x^{2}\;e^{-\cos(2\theta )x^{2}/10}\;dx\leq \frac{10}{\cos(2\theta )}\;e^{-\cos(2\theta )X^{2}/10}\left(X+\frac{5}{\cos(2\theta )X}\right)=:A_{X,\theta }$$

and we use the analytic expression *A*_*X*,*θ*_ to obtain a rigorous computable upper bound $A_{X,\theta }^{0}$ for *A*_*X*,*θ*_ and hence for *α*_*X*,*θ*_,

$$\alpha_{X,\theta }\leq A_{X,\theta }\leq A_{X,\theta }^{0}.$$

To this end, we first expand cos⁡(2θ) and use Taylor's theorem with remainder in Lagrange form to see that, for every m∈N,

3.4 $$\begin{array}{r} \cos(2\theta )\geq \sum_{j=0}^{4m}\frac{{\left(-1\right)}^{j}}{(2j)!}{\left(2\theta \right)}^{2j}=:T_{X,\theta }(m); \end{array}$$

note that $\cos^{\left(4m+1\right)}(x)=-\sin(x)\leq 0$ for every *x*∈[0,2*θ*]⊂[0,*π*/2]. If *θ* is a decimal fraction whose fractional part has three digits, the sum *T*_*X*,*θ*_(*m*) is rational and can be evaluated exactly. We choose a rigorous computable lower bound $T_{X,\theta }^{0}(m)$ of *T*_*X*,*θ*_(*m*) as the unique decimal number whose fractional part has six digits and $T_{X,\theta }^{0}(m)+10^{-6}>T_{X,\theta }(m)\geq T_{X,\theta }^{0}(m)$ (table 1). The function *f*(*t*):=(10/*t*) *e*^−*tX*^2^/10^(*X*+5/*tX*), *t*∈(0,1), is decreasing, hence, again by Taylor's theorem with remainder in Lagrange form, we obtain that, for all *m*, n∈N,

$$\begin{array}{rl} A_{X,\theta } & =f(\cos(2\theta ))\leq f(T_{X,\theta }^{0}(m))\\ & \leq \frac{10}{T_{X,\theta }^{0}(m)}\left(\sum_{k=0}^{2n+1}\frac{{\left(-1\right)}^{k}X^{2k}}{10^{k}k!}{\left(T_{X,\theta }^{0}(m)\right)}^{k}\right)\left(X+\frac{5}{T_{X,\theta }^{0}(m)X}\right)=:A_{X,\theta }(m,n). \end{array}$$

Now, we fix m,n∈N and proceed in the same way as for *T*_*X*,*θ*_(*m*) to obtain a rigorous computable upper bound $A_{X,\theta }^{0}(m,n)$ for *A*_*X*,*θ*_(*m*,*n*) with $A_{X,\theta }^{0}(m,n)-10^{-6}<A_{X,\theta }(m,n)\leq A_{X,\theta }^{0}(m,n)$ (table 1). Because *θ*↦*A*_*X*,*θ*_(*m*,*n*), *θ*∈[0,*π*/4), is increasing, the rigorous computable upper bound $A_{X,\theta_{0}}^{0}(m,n)$ for *θ*_0_:=0.75<*π*/4 is also an upper bound of *A*_*X*,*θ*_(*m*,*n*) for *θ*∈(0,*θ*_0_). Only in two of our computations (for the resonances numbered 37^−^ and 44^+^), we needed parameter values *θ* that are larger than *θ*_0_=0.75; their upper bound $A_{X,\theta }^{0}(m,n)$ is computed separately. We use *X*=50, *m*=2, *n*=32 and obtain the rigorous computable lower bounds $T_{X,\theta }^{0}(m)=:T_{X,\theta }^{0}$ and upper bounds $A_{X,\theta }^{0}(m,n)=:A_{X,\theta }^{0}$ displayed in table 1; note that for *X*=50 the condition $\cos(2\theta )\geq 0.8/x^{2}$ allows for $\theta \leq 0.5\arccos(\frac{8}{25}10^{-5})$, e.g. *θ*≤0.785238 very close to *π*/4∼0.7853981635.

**Table 1.** Rigorous computable bounds for *X*=50 and various *θ*∈[0,*π*/4).

for *θ*∈(0,0.75]:	$T_{X,\theta }^{0}=0.070737$	$A_{X,\theta }^{0}=0.000152$
for *θ*=0.755:	$T_{X,\theta }^{0}=0.060758$	$A_{X,\theta }^{0}=0.00216$

## 4. Guaranteed resonance enclosures and exclosures

**Figure 2.**
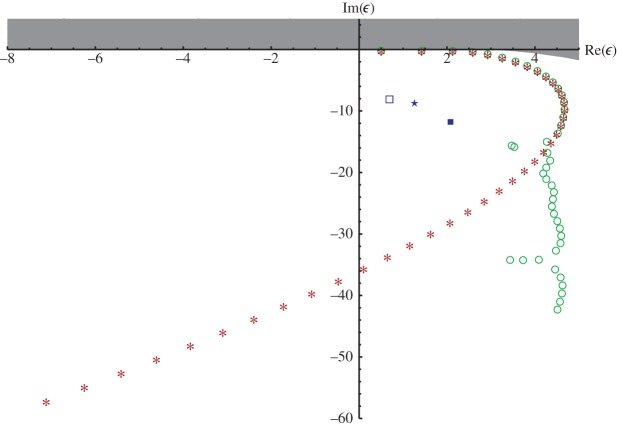
Resonance approximations computed by Rittby *et al.* (circles), Korsch *et al.* (asterisks), Abramov *et al.* (squares and star) and analytic bound from theorem 2.1. (Online version in colour.)

In [10], reply to comment, Rittby *et al.* listed a set of 44 approximate resonances $\epsilon_{k}^{\pm }$ of (1.1) that they computed numerically, along with a set of 40 approximate resonances claimed to be found numerically by Korsch *et al.* in [5], comment; here, the superscript + occurs for even *k*, whereas − occurs for odd *k*. The differences in modulus between these two approximate resonance strings are smaller than 2⋅10^−3^ up to $\epsilon_{15}^{-}$ and start to be larger than 10^−2^ from $\epsilon_{16}^{+}$ on, getting as huge as 56.19 for $\epsilon_{40}^{+}$ (figure 2).

We computed guaranteed enclosures for all 44 approximate resonances by Rittby *et al.* from [4] as well as exclosures for the approximate resonances $\epsilon_{16}^{+}$ up to $\epsilon_{28}^{+}$ by Korsch *et al.* from [5], comment. In addition, we enclosed the two pairs of resonances discovered numerically in [14] that are visible by the complex scaling method.

All computed enclosures for resonances, except for one of these pairs, were performed with interval length *X*=50, varying scaling angle *θ* as displayed in the tables, and corresponding guaranteed upper bound $A_{X,\theta }^{0}$ for *α*_*X*,*θ*_ as in table 1 at the end of §3. The enclosure for one of the additional resonance pairs in [14] turned out to be by far more challenging than all other computations.

We employ the interval arithmetic-based software library VNODE developed by Nedialkov *et al.* (see [15]) where all operations are performed with complex ‘intervals’, i.e. rectangles [*z*]=[*x*]+[*y*]*i*, where [*x*], [y]⊂R are closed intervals or singletons (see [16], §2 for a more detailed description). In the following, we use notation of the form

$$7.439759_{16958921987}^{70244416010}:=[7.43975916958921987,7.43975970244416010]$$

for intervals containing the real and imaginary part of resonances. Further, we use the enumeration $\epsilon_{k}^{\pm }$ to indicate the resonance number *k* and parity ± in the list of approximate resonances of Rittby *et al.* in [10, reply to comment, table I].

Note that the resonances coming from the boundary condition *y*(0)=0 have parity ‘−’, because the eigenfunctions of the corresponding eigenvalues of (2.4) are odd, whereas those coming from the boundary condition *y*′(0)=0 have parity ‘+’, because the eigenfunctions of the corresponding eigenvalues of (2.5) are even (see §2).

### (a) Guaranteed enclosures for resonance approximations by Rittby *et al.*

First, we present the computed enclosures for the 44 resonances $\lambda_{k}^{\pm }$ of problem (2.1) corresponding to the resonances $\epsilon_{k}^{\pm }$ listed in [10], reply to comment, table I.

Table 2 contains the enclosures for resonances λ=*e*^−2i*θ*^*z* via enclosures for eigenvalues *z* of (2.2) restricted to [0,∞) with Dirichlet boundary condition *y*(0)=0, i.e. eigenvalues of problem (2.4); table 3 contains the corresponding enclosures using eigenvalues *z* of (2.2) restricted to [0,∞) with Neumann boundary condition *y*′(0)=0, i.e. for eigenvalues of problem (2.5). Table 4 contains the enclosures for the 44 resonances $\epsilon_{k}^{\pm }=(\lambda_{k}^{\pm }+1.6)/2$ of the original problem (1.1) arising from the two sets of resonances $\lambda_{k}^{\pm }$ of (2.2) displayed in tables 2 and 3.

**Table 2.** Resonances for (2.2) from (2.4) on [0,∞) with *y*(0)=0.

$\left[\lambda_{1}^{-}\right]$	$1.241941_{860188297}^{939327957}$	$-0.0001165_{922328864033}^{148883330858}i$	*θ*=0.5
$\left[\lambda_{3}^{-}\right]$	$3.569165_{67301207}^{806997311}$	$-0.347501_{455537742}^{321552502}i$	*θ*=0.45
$\left[\lambda_{5}^{-}\right]$	$4.910972_{434212462}^{56906802}$	$-2.223063_{240885096}^{106029525}i$	*θ*=0.4
$\left[\lambda_{7}^{-}\right]$	$6.04865_{8993742452}^{9073751407}$	$-4.974892_{410423611}^{330291548}i$	*θ*=0.4
$\left[\lambda_{9}^{-}\right]$	$6.899910_{079540637}^{161900769}$	$-8.366339_{340877058}^{258516893}i$	*θ*=0.5
$\left[\lambda_{11}^{-}\right]$	$7.45761_{8940126859}^{9020283418}$	$-12.309461_{30179014}^{22163353}i$	*θ*=0.55
$\left[\lambda_{13}^{-}\right]$	$7.723776_{761948670}^{835325416}$	$-16.751102_{35451528}^{28113849}i$	*θ*=0.65
$\left[\lambda_{15}^{-}\right]$	$7.688281_{098696593}^{167564834}$	$-21.652528_{91022707}^{84135879}i$	*θ*=0.7
$\left[\lambda_{17}^{-}\right]$	$7.4397597_{27511353}^{99931500}$	$-26.931640_{5592495}^{4341806}i$	*θ*=0.73
$\left[\lambda_{19}^{-}\right]$	$5.34207_{5847041748}^{6377864414}$	$-30.84463_{146838990}^{093756717}i$	*θ*=0.725
$\left[\lambda_{21}^{-}\right]$	$6.96683_{1216924642}^{2261722650}$	$-33.21458_{959948786}^{855468978}i$	*θ*=0.735
$\left[\lambda_{23}^{-}\right]$	$6.915830_{548014552}^{614883968}$	$-37.987895_{76198814}^{69511864}i$	*θ*=0.72
$\left[\lambda_{25}^{-}\right]$	$6.91656_{0941325323}^{9434487592}$	$-41.75293_{945384829}^{096068594}i$	*θ*=0.725
$\left[\lambda_{27}^{-}\right]$	$7.263855_{509305397}^{642011094}$	$-46.005638_{64262755}^{50992176}i$	*θ*=0.725
$\left[\lambda_{29}^{-}\right]$	$7.1713970_{20278418}^{92698572}$	$-50.640608_{42764009}^{30257113}i$	*θ*=0.73
$\left[\lambda_{31}^{-}\right]$	$7.41606_{6375657773}^{8956119763}$	$-55.41738_{945099529}^{729172278}i$	*θ*=0.73
$\left[\lambda_{33}^{-}\right]$	$7.595038_{774505170}^{871916306}$	$-60.2052079_{9147592}^{2373976}i$	*θ*=0.74
$\left[\lambda_{35}^{-}\right]$	$7.354328_{549919785}^{634934198}$	$-65.0217411_{9449661}^{2859191}i$	*θ*=0.745
$\left[\lambda_{37}^{-}\right]$	$5.26985_{1946138838}^{5985565125}$	$-68.0334_{4294010962}^{3890068321}i$	*θ*=0.755
$\left[\lambda_{39}^{-}\right]$	$7.31756_{1972525686}^{2040413600}$	$-71.126990_{16800836}^{04488124}i$	*θ*=0.75
$\left[\lambda_{41}^{-}\right]$	$7.6551306_{14901760}^{78573411}$	$-76.322137_{25197381}^{18830202}i$	*θ*=0.75
$\left[\lambda_{43}^{-}\right]$	$7.54176_{1418798806}^{2505004713}$	$-81.61068_{243534949}^{046531832}i$	*θ*=0.75

**Table 3.** Resonances for (2.2) from (2.5) on [0,∞) with *y*′(0)=0.

$\left[\lambda_{2}^{+}\right]$	$2.654394_{092835094}^{176821622}$	$-0.030894_{64975797340}^{56577144434}i$	*θ*=0.35
$\left[\lambda_{4}^{+}\right]$	$4.248843_{771879894}^{813059968}$	$-1.1295899_{76541343}^{35361269}i$	*θ*=0.5
$\left[\lambda_{6}^{+}\right]$	$5.514431_{624202591}^{752453071}$	$-3.511012_{170826048}^{042575560}i$	*θ*=0.55
$\left[\lambda_{8}^{+}\right]$	$6.510869_{364918580}^{433786815}$	$-6.597282_{116776595}^{047908355}i$	*θ*=0.7
$\left[\lambda_{10}^{+}\right]$	$7.2155450_{18333105}^{98489665}$	$-10.272906_{38654053}^{30638393}i$	*θ*=0.55
$\left[\lambda_{12}^{+}\right]$	$7.6261961_{08709429}^{85861521}$	$-14.470753_{13697016}^{05981802}i$	*θ*=0.6
$\left[\lambda_{14}^{+}\right]$	$7.74690_{5966973172}^{6068847760}$	$-19.141473_{98214909}^{88027448}i$	*θ*=0.75
$\left[\lambda_{16}^{+}\right]$	$7.59265_{6102498221}^{7204389647}$	$-24.28598_{528682110}^{418492963}i$	*θ*=0.7
$\left[\lambda_{18}^{+}\right]$	$6.93961_{3775608598}^{4828882919}$	$-29.61377_{609379499}^{504052061}i$	*θ*=0.73
$\left[\lambda_{20}^{+}\right]$	$5.463698_{436278557}^{503147965}$	$-31.299089_{45643974}^{38957026}i$	*θ*=0.72
$\left[\lambda_{22}^{+}\right]$	$7.09171_{3513412909}^{5717195734}$	$-35.73909_{940568757}^{720190466}i$	*θ*=0.7
$\left[\lambda_{24}^{+}\right]$	$6.7766621_{07796643}^{74666058}$	$-39.913828_{10780846}^{04093896}i$	*θ*=0.72
$\left[\lambda_{26}^{+}\right]$	$7.1641875_{27769781}^{94122646}$	$-43.794599_{31759526}^{25124230}i$	*θ*=0.725
$\left[\lambda_{28}^{+}\right]$	$7.217834_{296771199}^{827593876}$	$-48.285288_{93378272}^{40295994}i$	*θ*=0.725
$\left[\lambda_{30}^{+}\right]$	$7.26422_{0853959247}^{1117277859}$	$-53.03794_{509870568}^{483538696}i$	*θ*=0.73
$\left[\lambda_{32}^{+}\right]$	$7.532859_{772590297}^{837890213}$	$-57.809513_{70761888}^{64231886}i$	*θ*=0.735
$\left[\lambda_{34}^{+}\right]$	$7.563081_{137412970}^{906616847}$	$-62.59350_{947366904}^{883239546}i$	*θ*=0.735
$\left[\lambda_{36}^{+}\right]$	$6.577678_{639847483}^{703519130}$	$-67.9668690_{7952254}^{1585076}i$	*θ*=0.75
$\left[\lambda_{38}^{+}\right]$	$5.86009_{0527100523}^{5868261508}$	$-68.1042_{1052927093}^{0518810982}i$	*θ*=0.75
$\left[\lambda_{40}^{+}\right]$	$7.566452_{115996163}^{416558969}$	$-73.72882_{409019465}^{387699096}i$	*θ*=0.745
$\left[\lambda_{42}^{+}\right]$	$7.629471_{689847954}^{817191209}$	$-78.94320_{909751929}^{897017587}i$	*θ*=0.75
$\left[\lambda_{44}^{+}\right]$	$7.424570_{055864947}^{340663545}$	$-84.175934_{92887123}^{43734233}i$	*θ*=0.755

**Table 4.** Resonances for (1.1).

	guaranteed enclosures		numerical values by Rittby *et al.*
$\epsilon_{1}^{-}$	$1.4209709_{300941485}^{696639785}$	$-0.0000582_{9611644320165}^{5744416654290}i$	1.420971−0.00005826663*i*
$\epsilon_{2}^{+}$	$2.1271970_{464175470}^{884108110}$	$-0.015447_{324878986700}^{282885722170}i$	2.127197−0.01544732*i*
$\epsilon_{3}^{-}$	$2.584582_{836506035}^{903498655}$	$-0.173750_{7277688710}^{6607762510}i$	2.584583−0.1737507*i*
$\epsilon_{4}^{+}$	$2.924421_{8859399470}^{9065299840}$	$-0.5647949_{882706715}^{676806345}i$	2.924422−0.564795*i*
$\epsilon_{5}^{-}$	$3.2554862_{171062310}^{845340100}$	$-1.111531_{6204425480}^{5530147625}i$	3.255486−1.111531*i*
$\epsilon_{6}^{+}$	$3.5572158_{121012955}^{762265355}$	$-1.7555060_{854130240}^{212877800}i$	3.557216−1.755506*i*
$\epsilon_{7}^{-}$	$3.824329_{4968712260}^{5368757035}$	$-2.487446_{2052118050}^{1651457740}i$	3.824330−2.487446*i*
$\epsilon_{8}^{+}$	$4.055434_{6824592900}^{7168934075}$	$-3.2986410_{583882975}^{239541775}i$	4.055435−3.298641*i*
$\epsilon_{9}^{-}$	$4.2499550_{397703185}^{809503845}$	$-4.1831696_{704385290}^{292584465}i$	4.249955−4.183170*i*
$\epsilon_{10}^{+}$	$4.4077725_{091665525}^{492448325}$	$-5.1364531_{93270265}^{53191965}i$	4.407773−5.136453*i*
$\epsilon_{11}^{-}$	$4.528809_{4700634295}^{5101417090}$	$-6.1547306_{50895070}^{10816765}i$	4.528809−6.154731*i*
$\epsilon_{12}^{+}$	$4.6130980_{543547145}^{929307605}$	$-7.2353765_{68485080}^{29909010}i$	4.613098−7.235377*i*
$\epsilon_{13}^{-}$	$4.661888_{3809743350}^{4176627080}$	$-8.3755511_{77257640}^{40569245}i$	4.661888−8.375551*i*
$\epsilon_{14}^{+}$	$4.67345_{29834865860}^{3034423880}$	$-9.5707369_{91074545}^{40137240}i$	4.673453−9.570737*i*
$\epsilon_{15}^{-}$	$4.6441405_{493482965}^{837824170}$	$-10.8262644_{55113535}^{20679395}i$	4.644141−10.82626*i*
$\epsilon_{16}^{+}$	$4.596328_{0512491105}^{6021948235}$	$-12.142992_{643410550}^{092464815}i$	4.596328−12.14299*i*
$\epsilon_{17}^{-}$	$4.5198798_{637556765}^{999657500}$	$-13.4658202_{79624750}^{17090300}i$	4.519880−13.46582*i*
$\epsilon_{18}^{+}$	$4.26980_{68878042990}^{74144414595}$	$-14.80688_{8046897495}^{7520260305}i$	4.269807−14.80689*i*
$\epsilon_{19}^{-}$	$3.47103_{79235208740}^{81889322070}$	$-15.422315_{734194950}^{468783585}i$	3.471038−15.42232*i*
$\epsilon_{20}^{+}$	$3.5318492_{181392785}^{515739825}$	$-15.649544_{728219870}^{694785130}i$	3.531849−15.64954*i*
$\epsilon_{21}^{-}$	$4.28341_{56084623210}^{61308613250}$	$-16.607294_{799743930}^{277344890}i$	4.283416−16.60729*i*
$\epsilon_{22}^{+}$	$4.34585_{67567064545}^{78585978670}$	$-17.86954_{9702843785}^{8600952330}i$	4.345857−17.86955*i*
$\epsilon_{23}^{-}$	$4.257915_{2740072760}^{3074419840}$	$-18.9939478_{80994070}^{47559320}i$	4.257915−18.99395*i*
$\epsilon_{24}^{+}$	$4.1883310_{538983215}^{873330290}$	$-19.9569140_{53904230}^{20469480}i$	4.188331−19.95691*i*
$\epsilon_{25}^{-}$	$4.25828_{04706626615}^{47172437960}$	$-20.87646_{9726924145}^{5480342970}i$	4.258283−20.87647*i*
$\epsilon_{26}^{+}$	$4.3820937_{638848905}^{970613230}$	$-21.8972996_{58797630}^{25621150}i$	4.382094−21.89730*i*
$\epsilon_{27}^{-}$	$4.431927_{7546526985}^{8210055470}$	$-23.002819_{321313775}^{254960880}i$	4.431928−23.00282*i*
$\epsilon_{28}^{+}$	$4.408917_{1483855995}^{4137969380}$	$-24.142644_{466891360}^{201479970}i$	4.408918−24.14264*i*
$\epsilon_{29}^{-}$	$4.3856985_{101392090}^{463492860}$	$-25.320304_{2138200450}^{1512855650}i$	4.385699−25.32030*i*
$\epsilon_{30}^{+}$	$4.432110_{4269796235}^{5586389295}$	$-26.518972_{549352840}^{417693480}i$	4.432110−26.51897*i*
$\epsilon_{31}^{-}$	$4.50803_{31878288865}^{44780598815}$	$-27.70869_{4725497645}^{3645861390}i$	4.508034−27.70869*i*
$\epsilon_{32}^{+}$	$4.566429_{8862951485}^{9189451065}$	$-28.9047568_{53809440}^{21159430}i$	4.566430−28.90476*i*
$\epsilon_{33}^{+}$	$4.597519_{3872525850}^{4359581530}$	$-30.1026039_{95737960}^{61869880}i$	4.597520−30.10260*i*
$\epsilon_{34}^{+}$	$4.581540_{5687064850}^{9533084235}$	$-31.296754_{736834520}^{416197730}i$	4.581541−31.29675*i*
$\epsilon_{35}^{+}$	$4.477164_{2749598925}^{3174670990}$	$-32.5108705_{97248305}^{64295955}i$	4.477164−32.51087*i*
$\epsilon_{36}^{+}$	$4.0888393_{199237415}^{517595650}$	$-33.9834345_{39761270}^{07925380}i$	4.088839−33.98343*i*
$\epsilon_{37}^{+}$	$3.43492_{59730694190}^{79927825625}$	$-34.0167_{21470054810}^{19450341605}i$	3.434927−34.01672*i*
$\epsilon_{38}^{+}$	$3.73004_{52635502615}^{79341307540}$	$-34.05210_{5264635465}^{2594054910}i$	3.730047−34.05210*i*
$\epsilon_{39}^{+}$	$4.45878_{09862628430}^{10202068000}$	$-35.5634950_{84004180}^{22440620}i$	4.458781−35.56350*i*
$\epsilon_{40}^{+}$	$4.583226_{0579980815}^{2082794845}$	$-36.86441_{2045097325}^{1938495480}i$	4.583226−36.86441*i*
$\epsilon_{41}^{+}$	$4.6275653_{074508800}^{392867055}$	$-38.161068_{625986905}^{594151010}i$	4.627565−38.16107*i*
$\epsilon_{42}^{+}$	$4.614735_{8449239770}^{9085956045}$	$-39.471604_{548759645}^{485087935}i$	4.614736−39.47160*i*
$\epsilon_{43}^{+}$	$4.57088_{07093994030}^{12525023565}$	$-40.80534_{1217674745}^{0232659160}i$	4.570881−40.80534*i*
$\epsilon_{44}^{+}$	$4.512285_{0279324735}^{1703317725}$	$-42.087967_{464435615}^{218671165}i$	4.512285−42.08797*i*

The enclosing boxes for the resonances *ϵ* of (1.1) are obtained from the enclosing boxes for the eigenvalues *z* of (2.2) as follows. If [*u*_1_,*u*_2_]+[*v*_1_,*v*_2_]*i* is an enclosing box in the *z*-plane, then the enclosing box [*x*_1_,*x*_2_]+[*y*_1_,*y*_2_]*i* for a resonance λ=e^−2i*θ*^*z* of (2.1) is the smallest axis-parallel box that contains the rotated box e^−2i*θ*^([*u*_1_,*u*_2_]+[*v*_1_,*v*_2_]*i*). The corresponding enclosing box for a resonance *ϵ*=(λ+1.6)/2 of (1.1) is obtained from

$$\lambda \in [x_{1},x_{2}]+[y_{1},y_{2}]i\;\;⟺\;\;\epsilon \in \left[\frac{x_{1}+1.6}{2},\frac{x_{2}+1.6}{2}\right]+\left[\frac{y_{1}}{2},\frac{y_{2}}{2}\right]i.$$

The values of the 44 approximate resonances of (1.1) listed in [10], reply to comment, table I, which were computed by Rittby *et al.* in floating point arithmetic without error bounds, are displayed in the right column in table 4; they agree with our enclosures at least up to order 10^−4^. Thus, our guaranteed enclosures prove that all values computed by Rittby *et al.* do indeed lie near true resonances.

### (b) Guaranteed exclosures for resonance approximations by Korsch *et al.*

On the other hand, we applied our method to the numerical values of the resonance approximations of Korsch *et al.* numbered 16^+^,17^−^,…,27^−^, 28^+^ in [10], reply to comment, table II; note that the resonance approximations 29^−^,…,40^+^ therein can not be seen by the complex scaling method.

Using larger boxes around these numerical values, we found that in each case the interval-valued argument principle yields an interval [*c*_1_,*c*_2_] with $\left[c_{1},c_{2}]\cap N_{0}=\{0\right\}$, which proves that there are no eigenvalues in the considered box (see (1.2)). The box side lengths *l*_*k*_∈[0.1,2] are listed in table 5. For every resonance approximation $\epsilon_{k}^{\pm },$ the corresponding approximate value in the *z*-plane is denoted by $z_{k}^{\pm }$. The midpoint $M_{k}\in C$ of the box with side length *l*_*k*_ in the *z*-plane is chosen such that

|Re(Mk)−Re(zk±)|<0.05≤lk2,|Im(Mk)−Im(zk±)|<0.05≤lk2.

**Figure 3.**
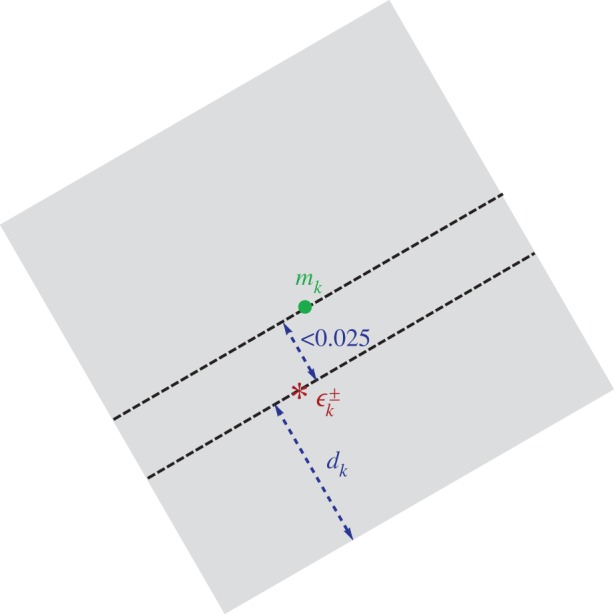
Rotated box of side length *l*_*k*_/2 excluding approximate resonance $\epsilon_{k}^{\pm }$, *k*=16^+^,…,28^+^, from Korsch *et al.* marked by asterisk. (Online version in colour.)

The corresponding box in the *ϵ*-plane is scaled and rotated owing to the relation $\epsilon_{k}^{\pm }=(e^{-2i\theta }z_{k}^{\pm }+1.6)/2$. The box has side length *l*_*k*_/2 and is rotated clockwise by the angle 2*θ* around the midpoint *m*_*k*_:=(e^−2i*θ*^*M*_*k*_+1.6)/2. The minimal distance *d*_*k*_ of $\epsilon_{k}^{\pm }$ to the boundary of the rotated box satisfies *d*_*k*_>(*l*_*k*_/4)−0.025≥0 (figure 3).

**Table 5.** Excluded resonances.

numerical values by Korsch *et al.*		chosen box side length *l*_*k*_ and angle *θ*	
$\left[\epsilon_{16}^{+}\right]$	4.589120−12.13151*i*	*l*_16_=0.1	*θ*=0.7
$\left[\epsilon_{17}^{-}\right]$	4.493625−13.49000*i*	*l*_17_=0.1	*θ*=0.73
$\left[\epsilon_{18}^{+}\right]$	4.362774−14.89927*i*	*l*_18_=0.6	*θ*=0.73
$\left[\epsilon_{19}^{-}\right]$	4.196770−16.35807*i*	*l*_19_=1.0	*θ*=0.725
$\left[\epsilon_{20}^{+}\right]$	3.995807−17.86525*i*	*l*_20_=1.4	*θ*=0.72
$\left[\epsilon_{21}^{-}\right]$	3.760076−19.41977*i*	*l*_21_=1.5	*θ*=0.72
$\left[\epsilon_{22}^{+}\right]$	3.489755−21.02065*i*	*l*_22_=0.5	*θ*=0.725
$\left[\epsilon_{23}^{-}\right]$	3.185024−22.66701*i*	*l*_23_=1.4	*θ*=0.725
$\left[\epsilon_{24}^{+}\right]$	2.846045−24.35801*i*	*l*_24_=2.0	*θ*=0.725
$\left[\epsilon_{25}^{-}\right]$	2.472984−26.09287*i*	*l*_25_=2.0	*θ*=0.725
$\left[\epsilon_{26}^{+}\right]$	2.065991−27.87087*i*	*l*_26_=2.0	*θ*=0.72
$\left[\epsilon_{27}^{-}\right]$	1.625219−29.69132*i*	*l*_27_=2.0	*θ*=0.725
$\left[\epsilon_{28}^{+}\right]$	1.150811−31.55357*i*	*l*_28_=2.0	*θ*=0.725

Hence, our guaranteed exclosures prove that none of the numerical values of Korsch *et al.* numbered 16^+^,17^−^,…,27^−^,28^+^ lies near a true resonance of (1.1).

### (c) Enclosures of resonance approximations by Abramov *et al.*

Finally, we considered the three pairs of additional resonances found in [14, p. 72], one pair near each of the points

$${\hat{\epsilon }}_{1}=0.69-7.91i,\;{\hat{\epsilon }}_{2}=1.26-8.51i,\;{\hat{\epsilon }}_{3}=2.08-11.61i;$$

the corresponding values λ=2*ϵ*−1.6 are

$${\hat{\lambda }}_{1}=-0.22-15.82i,\;{\hat{\lambda }}_{2}=0.92-17.02i,\;{\hat{\lambda }}_{3}=2.46-23.22i.$$

These new resonances were conjectured to exist not by means of complex scaling, but by exploiting the asymptotic properties of the solution of a differential equation with a rapidly decaying potential; numerical methods were developed to locate these resonances (see [14], §6). In fact, the method of complex scaling does not allow one to see the first pair of resonances near ${\hat{\lambda }}_{1}$ because it has negative real part, but it does allow one to see the second and third pair near ${\hat{\lambda }}_{2}$, ${\hat{\lambda }}_{3}$.

We computed guaranteed enclosures for the two pairs of resonances near ${\hat{\lambda }}_{2}$, ${\hat{\lambda }}_{3}$; each of these pairs originates in one eigenvalue *z* of (2.4) with boundary condition *y*(0)=0 with odd eigenfunction (denoted by superscript ‘−’) and one eigenvalue *z* of (2.5) with boundary condition *y*′(0)=0 with even eigenfunction (denoted by superscript ‘+’). The guaranteed enclosures we obtained for the four resonances ${\hat{\lambda }}_{2}^{-}$, ${\hat{\lambda }}_{2}^{+}$, ${\hat{\lambda }}_{3}^{-}$, ${\hat{\lambda }}_{3}^{+}$ are shown in table 6.

**Table 6.** Enclosures of resonance pairs near ${\hat{\lambda }}_{2}$, ${\hat{\lambda }}_{3}$ computed by Abramov *et al.* [14].

$\left[{\hat{\lambda }}_{2}^{-}\right]$	$0.91_{7362166176397}^{8411650805308}$	$-17.00_{1197218127467}^{0249282468423}i$	*θ*=0.76
$\left[{\hat{\lambda }}_{2}^{+}\right]$	$0.91_{7362166176397}^{8411650805308}$	$-17.00_{1197218127467}^{0249282468423}i$	*θ*=0.76
$\left[{\hat{\lambda }}_{3}^{-}\right]$	$2.55_{6817300474516}^{7790013021589}$	$-23.21_{306897699860}^{200893060222}i$	*θ*=0.735
$\left[{\hat{\lambda }}_{3}^{+}\right]$	$2.560_{054480374655}^{188212981834}$	$-23.208_{57870390974}^{44496992380}i$	*θ*=0.735

The computation of the resonance pair ${\hat{\lambda }}_{3}^{-}$, ${\hat{\lambda }}_{3}^{+}$ was performed in the same way as the enclosures described in §4*a*. Choosing *θ*=0.735, our provably correct computations showed that for each of the two boundary conditions there is only one resonance ${\hat{\lambda }}_{3}^{-}$ and ${\hat{\lambda }}_{3}^{+}$, respectively, in the disjoint boxes displayed in table 6. Moreover, they guarantee that in the larger λ-box e^−2i*θ*^([23,24]+[0.05,1]*i*) containing these two boxes as well as the numerical value ${\hat{\lambda }}_{3}$ of Abramov *et al.* there is only one resonance for each of the two boundary conditions. Altogether, we thus proved that there is precisely one pair of disjoint resonances ${\hat{\lambda }}_{3}^{-}\neq {\hat{\lambda }}_{3}^{+}$ near the resonance approximation ${\hat{\lambda }}_{3}=2.46-23.22i$ of Abramov *et al.* and that this approximation has distance approximately 1⋅10^−1^ to the true resonance pair ${\hat{\lambda }}_{3}^{\pm }$.

The computation of the resonance pair ${\hat{\lambda }}_{2}^{-}$, ${\hat{\lambda }}_{2}^{+}$ turned out to be much harder and computationally more expensive than all other enclosures and exclosures. To make it work, we had to use a slight modification of usual complex scaling, using stretching by some parameter *R*>0 in addition to rotation of the variable by an angle *θ*∈[0,*π*/4). The potential *q*_*θ*,*R*_ and the eigenvalue parameter *z* in the spectral problem for the corresponding operator *H*_*θ*,*R*_ (compare (2.2), (2.3)) then become

$$q_{\theta ,R}(x):=R^{2}\;e^{2i\theta }(R^{2}\;e^{2i\theta }x^{2}-1.6)\;e^{-R^{2}\;e^{2i\theta }x^{2}/10},\;x\in R,\;z:=R^{2}\;e^{2i\theta }\lambda ;$$

note that usual complex scaling corresponds to *R*=1.

In order to apply Levinson's theorem, we needed to find suitable *X*≥0, *θ*∈[0,*π*/4) and *R*>0 such that $\alpha_{X,\theta ,R}:=\int_{X}^{\infty }|q_{\theta ,R}(x)|\;dx$ satisfies *α*_*X*,*θ*,*R*_<1. Proceeding as for usual complex scaling, instead of (3.3), we used

$$\alpha_{X,\theta ,R}\leq \frac{10R^{2}}{\cos(2\theta )}\;e^{-R^{2}\cos(2\theta )X^{2}/10}\left(X+\frac{1}{R^{2}}\frac{5}{\cos(2\theta )X}\right)=:A_{X,\theta ,R}.$$

The main benefit of the additional stretching is that the upper bound *A*_*X*,*θ*,*R*_ decays exponentially fast in *R*. As for usual complex scaling, we then applied Taylor's theorem with remainder in Lagrange form to obtain the rigorous computable upper bound $A_{X,\theta ,R}^{0}=1.77\cdot 10^{-17}$ for *X*=10, *θ*=0.76 and *R*=10.

With these parameters, we succeeded to enclose the resonances ${\hat{\lambda }}_{2}^{-}$, ${\hat{\lambda }}_{2}^{+}$ for the boundary condition *y*(0)=0 and *y*′(0)=0, respectively. The corresponding values in the *z*-plane are both in the box $1702._{49}^{59}+5._{3}^{4}i$, hence

$${\hat{\lambda }}_{2}^{-},\;{\hat{\lambda }}_{2}^{+}\in R^{-2}\;e^{-2i\theta }(1702._{49}^{59}+5._{3}^{4}i)\subset 0.91_{7362166176397}^{8411650805308}-17.00_{1197218127467}^{0249282468423}i.$$

Here, the first set is a box with midpoint *R*^−2^ e^−2i*θ*^(1702.54+5.35*i*)≈0.918−17.001*i* and side length *R*^−2^10^−1^=1⋅10^−3^, rotated clockwise by the angle 2*θ*=1.52; the second set, which is the one displayed in table 6, is the smallest axis-parallel box containing this rotated box. Note that these enclosures for ${\hat{\lambda }}_{2}^{\pm }$ differ in modulus by approximately 2⋅10^−2^ from the value ${\hat{\lambda }}_{2}=0.92-17.02i$ calculated by Abramov *et al.* [14].

Hence, our guaranteed enclosures prove that not far from each of the two numerically computed values ${\hat{\lambda }}_{2}$ and ${\hat{\lambda }}_{3}$ of Abramov *et al.* there is indeed a pair of true resonances of (2.1); the distance is approximately 2⋅10^−2^ for ${\hat{\lambda }}_{2}$ and approximately 1⋅10^−1^ for ${\hat{\lambda }}_{3}$.

## 5. Conclusion

In this paper, we have presented a method which, for the first time, permits one to compute resonances in atomic physics with absolute certainty. At the same time, it allows one to detect with absolute certainty wrongly computed resonance approximations. The absolute reliability of our approach is based on a combination of interval arithmetic and the argument principle. To prove the efficiency of our method, we have established guaranteed *en*closures for all numerical resonance approximations of Rittby *et al.* in [4,7] for problem (1.1) and guaranteed *ex*closures for the numerically computed values of Korsch *et al.* in [5] that are visible to complex scaling, thus definitely settling a dispute between these two groups of authors. The greatest challenge was to provably enclose two additional pairs of approximate resonances computed by Abramov *et al.* in [14] that were found neither by Rittby *et al.* nor by Korsch *et al.* Thus, we have proved the conjecture in [4,7] that the real parts of auto-ionizing resonances of certain atoms and molecules exhibit an oscillatory behaviour beyond a threshold and we have added new information on this threshold originating in the two new confirmed pairs of resonances.

**Figure 4.**
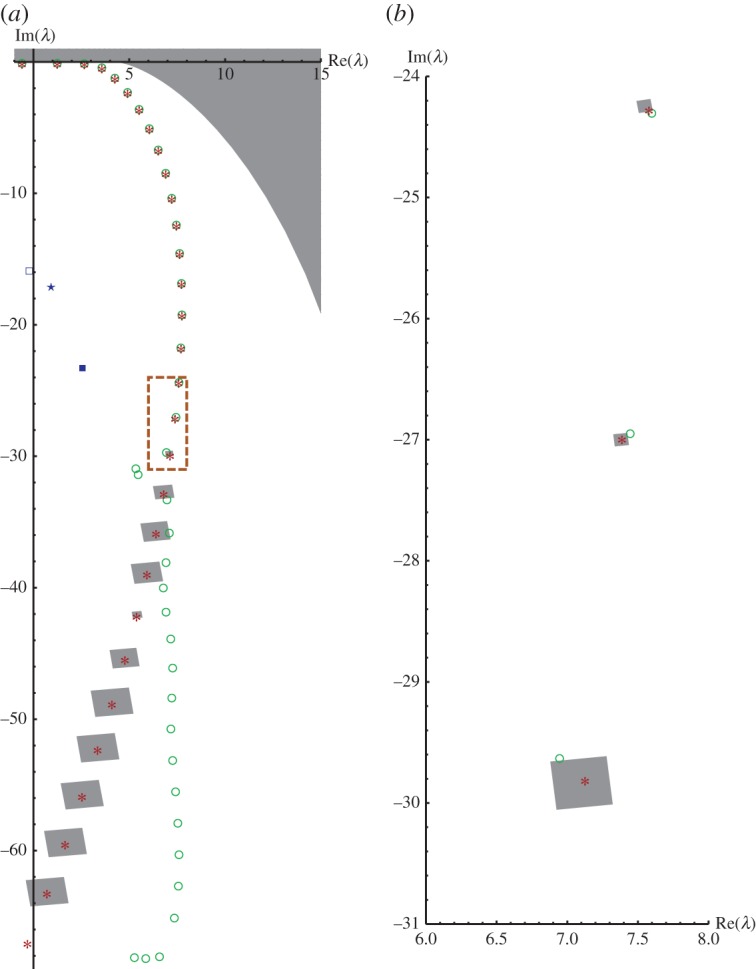
Excluded approximate resonances of Korsch *et al.* (asterisks) surrounded by respective excluding boxes, together with approximate resonances of Rittby *et al.* (circles), Abramov *et al.* (star and square) and analytic exclosure (grey-shaded) in the λ-plane. (*a*) Approximate resonances 0^+^,…,38^+^ of Rittby *et al.*, 0^+^,…,29^−^ of Korsch *et al.* and excluding boxes for approximate resonances 16^+^,…,28^+^ by Korsch *et al*. (*b*) Zoom into region marked by dashed line in (*a*) showing the approximate resonances 16^+^,17^−^,18^+^ with respective excluding boxes. (Online version in colour.)

Figure 4*a* displays all our results in the rectangle 0≤Re(λ)≤15, −70≤Im(λ)≤0: in the top right corner of the λ-plane, the analytic exclusion from theorem 2.1 (grey-shaded), the enclosed approximate resonances 1^−^,…,38^+^ of Rittby *et al.* surrounded by circles, the additional ones by Abramov *et al.* as star and square, and the claimed approximate resonances 1^−^,…,29^−^ of Korsch *et al.* as asterisks; note that the resonances 0^+^, 29^−^ and ${\hat{\lambda }}_{1}$ to the left of the imaginary axis cannot be seen by the complex scaling method because of their negative real part. Around every disproved approximate resonances 16^+^,…,28^+^ of Korsch *et al.,* our excluding box is shown (grey-shaded). Figure 4*b* illustrates that for resonance 16^+^ it was especially difficult to find a box that simultaneously *ex*cludes the computed value of Korsch *et al.* and does *not* contain the value computed by Rittby *et al.*
